# CXCL4 Plasma Levels Are Not Associated with the Extent of Coronary Artery Disease or with Coronary Plaque Morphology

**DOI:** 10.1371/journal.pone.0141693

**Published:** 2015-11-02

**Authors:** Christian Erbel, Grigorios Korosoglou, Pearlyn Ler, Mohammadreza Akhavanpoor, Gabriele Domschke, Fabian Linden, Andreas O. Doesch, Sebastian J. Buss, Evangelos Giannitsis, Hugo A. Katus, Christian A. Gleissner

**Affiliations:** 1 Department of Cardiology, University Hospital, Im Neuenheimer Feld 410, 69120, Heidelberg, Germany; 2 DZHK (German Centre for Cardiovascular Research), partner site Heidelberg, Germany; University of Glasgow, UNITED KINGDOM

## Abstract

**Background:**

CXCL4 is a platelet chemokine released at micromolar concentrations upon platelet activation. CXCL4 has been shown to promote atherogenesis by various mechanisms. However, data on CXCL4 plasma levels in patients with coronary artery disease are largely inconclusive. Computed coronary artery angiography (CCTA) represents an excellent tool to quantify and characterize coronary atherosclerotic plaques. We hypothesized that increased CXCL4 plasma levels may be associated with features of plaque instability resulting in adverse cardiovascular events. Specifically, we sought to determine whether CXCL4 levels are correlated with specific features of coronary artery disease including (1) plaque volume, (2) calcium score, (3) degree of stenosis, or (4) vascular remodeling.

**Methods and Results:**

CXCL4 plasma levels were measured by ELISA in 217 patients undergoing CCTA for suspected CAD (mean age 64.2 ± 9.4 years, 107 (49.3%) male). Mean CXCL4 plasma levels were 12.5 ± 4.6 ng/mL. There was no significant correlation between CXCL4 levels and any clinical or demographic parameters including cardiovascular risk factors. CXCL4 plasma levels did not differ between patient with or without coronary artery disease (CAD: 12.5 ± 4.5 ng/ml, no CAD: 12.5 ± 4.8 ng/ml). Neither univariate nor multivariate analysis showed an association between CXCL4 levels and plaque volume, total calcium score, degree of stenosis, or vascular remodeling. Subgroup analysis of patients with CAD as confirmed by CCTA did not show any association of CXCL4 levels with the extent of CAD.

**Conclusions:**

While CXCL4 may be present and active within the arterial wall, local increase of CXCL4 may not translate into systemically elevated CXCL4 levels. Further studies will have to test whether CXCL4 may still represent a suitable therapeutic target in human atherosclerosis.

## Introduction

Atherosclerosis and its consequences such as myocardial infarction and stroke remain the major cause of mortality worldwide [1]. As an inflammatory disease of the arterial wall, atherosclerotic lesion formation is promoted by inflammatory cells and their pro-inflammatory mediators. Apart from blood-borne monocytes, monocyte-derived cells, T and B cells within the subendothelial space, activated platelets play an important role in the development of atherosclerotic lesions [2–5]. Platelets not only mediate blood coagulation and thrombus formation, but they are a rich source of chemokines which are secreted upon platelet activation [6,7].

CXCL4 (formerly known as platelet factor-4) is one of the most abundant platelet chemokines and is released in micromolar concentrations from the platelets’ alpha granula upon platelet activation [8]. In *Apoe* ^-/-^ mice, the genetic deletion of the *Pf4* gene coding for CXCL4 results in advanced atherosclerotic lesion formation [9]. CXCL4 is known to affect atherosclerotic lesion development at various stages. In conjunction with CCL5, it forms heterodimers that may be deposited at the vascular endothelial surface and mediate monocyte recruitment to the vascular wall [10]. Furthermore, CXCL4 may promote monocyte macrophage differentiation resulting in a specific macrophage subtype termed M4 [11,12]. Clinically, the presence of CXCL4 within carotid endarterectomy specimens could be shown to be associated with clinical symptoms [13]. Similarly, we could recently show that the presence of M4 macrophage within human atherosclerotic lesions is associated with advanced plaque morphology [14,15]. Accordingly, there is good evidence suggesting a vital for CXCL4 in atherogenesis and progression of atherosclerosis.

Previous studies investigating the role of CXCL4 plasma levels in coronary artery disease have lead to conflicting results: While some studies found that elevated CXCL4 plasma levels were associated with CAD (but not with acute myocardial infarction) [16], others did not detect any differences [17]. The underlying reasons for these divergent findings are not entirely resolved. Based on the evidence clearly suggesting a pro-atherogenic role for CXCL4 and the availability of novel imaging tools that allow detailed characterization of coronary atherosclerotic plaque composition such as coronary computed tomography angiography (CCTA) [18], we sought to determine whether CXCL4 levels are related with specific features of coronary artery disease including (1) plaque volume, (2) calcium score, (3) degree of stenosis, or (4) vascular remodeling. To this end, CXCL4 plasma levels were measured in 217 in patients with suspected coronary artery disease undergoing cardiac computed tomography angiography.

## Materials and Methods

### Study population

The study population comprised 217 patients with preserved LV-function, undergoing clinically indicated coronary computed tomography angiography (CCTA) for suspected coronary artery disease (CAD). The clinical presentation of patients referred for CCTA included exercise-induced chest pain (16%), chest pain at rest (25%), dyspnoea (35%), or other including non-specific symptoms or positive treadmill testing (24%).

With approval from the local ethics committee at the Department of Cardiology, University of Heidelberg, Heidelberg/Germany (IRB protocol S-317/2008), blood was drawn at the time of CCTA after obtaining written informed consent for participation in our study. All studies were performed in accordance with the ethical standards laid down in the 1964 Declaration of Helsinki and its later amendments.

Cardiovascular risk factors were recorded at the time of the CCTA. They were defined as arterial hypertension (blood pressure ≥140/90 mm Hg or antihypertensive therapy) [19], hyperlipidemia (low-density lipoprotein cholesterol (LDL-C) ≥130 mg/dL or statin therapy) [20], current or prior smoking, diabetes mellitus, and a family history of CAD.

### 256-slice CT scanning technique

CCTA was performed using a 256-slice Brilliance iCT scanner (Philips Healthcare, Hamburg, Germany) that features a gantry rotation time of 270 ms, resulting in a temporal resolution of 36–135 ms, depending on the heart rate of the patient, and an isotropic sub-millimetre spatial resolution of 0.67×0.67×0.67 mm^3^. The CCTA protocol and quantitative assessment of atherosclerotic plaque components is described in detail elsewhere [21,22]. Patients without any plaque or stenosis were considered patients „without CAD“.

### ELISA

CXCL4 plasma levels were measured in duplicates in a blinded fashion using the RayBio Human PF-4 ELISA Kit (RayBiotech, Norcross/USA) according to the manufacturer’s instructions.

### Statistical analysis

Statistical analyses were performed using Prism (GraphPad, La Jolla/USA) and SPSS (IBM, Ehningen/ Germany). Sample size and power calculation was performed using G*Power (www.gpower.hhu.de).

Based on the sample size of 217 individuals (110 with CAD as detected by CCTA, 107 without CAD), a significance criterion of α = 0.05, and a power of 0.8, the study was powered detect a small to medium effect with an effect size of d = 0.37 as defined previously [23], which is reasonable to detect clinically meaningful differences between patients with and without CAD.

P values were two-tailed, P<0.05 was considered significant. Continuous data are presented as means ± standard deviations, categorical data are presented as absolute numbers and percentages. Normal distribution was tested for by d’Agostino Pearson omnibus normality testing. Correlation was determined by calculating Spearman’s rank correlation coefficient. Means were compared using t-test. For logistic regression analysis, data lacking normal distribution were log-transformed. Cardiovascular risk factors were included as one parameter comprising gender, hypertension, hyperlipidemia, diabetes, family history for cardiovascular disease, and history of tobacco use.

Intra- and interobserver variability for quantification of plaque volume and observer agreement for the presence or absence of vascular remodeling were calculated by repeated analysis of 40 randomly selected cases. As described previously, the readings were separated by 8 weeks to minimize recall bias [22].

## Results

### Patient characteristics

Overall, 217 patients were studied (mean age 64.2 ± 9.4 years, 107 (49.3%) male, Table 1). On average, patients displayed 2.3 ± 1.4 cardiovascular risk factors indicating an intermediate risk for coronary artery disease (CAD). In terms of medication, about 40% of patients received platelet inhibitors, β blockers, ACE-inhibitors/angiotensin receptor blockers, and statins. High sensitivity troponin T (hs-TnT) levels were below the pathological threshold (14 pg/mL for hs-TnT). High sensitivity C-reactive protein levels were slightly increased (pathological threshold 0.4 mg/L).

**Table 1:** Patient characteristics. Demographic and clinical data of all patients and divided by CXCL4 quartiles. For each parameter the coefficient and P value with CXCL4 plasma levels is indicated as calculated by Pearson’s correlation. Continuous variables are indicated as mean ± standard deviation, categorical variables are indicated as absolute number (percentage).

Parameter	All patients (n = 217)	1^st^ quartile (n = 50)	2^nd^ quartile (n = 57)	3^rd^ quartile (n = 55)	4^th^ quartile (n = 57)	Correlation coefficient with CXCL4 levels	P value
**Demographics**							
Age (years)	64.2 ± 9.4	67.0 ± 8.3	63.8 ± 10.3	61.4 ± 9.7	64.8 ± 8.4	-0.083	0.241
Male sex	107 (49.3)	23 (46.0)	30 (52.6)	27 (49.1)	27 (49.1)	0.011	0.873
**Coronary risk factors**							
1. Arterial hypertension	153 (70.5)	37 (74.0)	42 (73.7)	37 (67.3)	37 (67.3)	-0.037	0.613
2. Hypercholesterolemia	122 (56.2)	32 (64.0)	32 (56.1)	30 (54.5)	28 (50.9)	-0.072	0.313
3. Diabetes mellitus	15 (6.9)	4 (8.0)	3 (5.3)	4 (7.3)	4 (7.3)	-0.030	0.864
4. Family history of coronary artery disease	73 (33.6)	18 (36.0)	19 (33.3)	21 (38.2)	15 (27.3)	-0.150	0.061
5. Smoking	64 (29.5)	10 (20.0)	22 (38.6)	17 (30.9)	15 (27.3)	0.029	0.684
6. Obesity	27 (12.4)	10 (20.0)	7 (12.3)	5 (9.1)	5 (9.1)	-0.114	0.153
Total number of risk factors (0–5)	2.3 ± 1.4	2.5 ± 1.7	2.4 ± 1.3	2.3 ±1.3	2.1 ± 1.5	-0.128	0.059
**Cardiac medications**							
Aspirin (100 mg/day) or clopidogrel (75 mg/day)	85 (39.2)	17 (34.0)	25 (43.9)	23 (41.1)	20 (40.0)	0.043	0.563
Coumadin	13 (6.0)	7 (14.0)	0 (0.0)	1 (1.8)	5 (9.1)	-0.036	0.635
β-blocker	96 (44.2)	29 (58.0)	24 (42.1)	21 (37.5)	22 (40.0)	-0.085	0.441
ACE inhibitor or angiotensin receptor blocker	81 (37.3)	20 (40.0)	23 (40.4)	19 (34.5)	19 (34.5)	-0.86	0.262
Statin	83 (38.2)	23 (46.0)	25 (43.9)	18 (32.7)	17 (30.9)	-0.058	0.441
Nitrates	4 (1.8)	1 (2.0)	1 (1.8)	0 (0)	2 (3.6)	0.169	0.073
**Laboratory data**							
CXCL4 (μg/mL)	12.5 ± 4.6	6.7 ± 2.9	10.8 ± 0.8	13.8 ± 0.8	18.1 ± 2.7	-	-
High sensitivity CRP (mg/L)	3 ± 3	2 ± 0	5 ± 7	2 ± 0	2 ± 1	-0.16	0.922
High sensitivity troponin T (pg/mL)	7 ± 8	9 ± 10	4 ± 2	6 ± 7	9 ± 10	0.072	0.565

### CXCL4 levels and cardiovascular risk factors

Mean CXCL4 plasma levels were 12.5 ± 4.6 ng/mL. There was no significant correlation between CXCL4 levels and any clinical or demographic parameters. Notably, CXCL4 levels were did not correlate with any cardiovascular risk factor. Also, there was no correlation with intake of platelet inhibitors. A trend was seen for an inverse correlation between CXCL4 levels and the total number of cardiovascular risk factors, however this was not significant (r = -0.128, P = 0.059).

### CXCL4 levels and coronary artery disease

**Fig 1:**
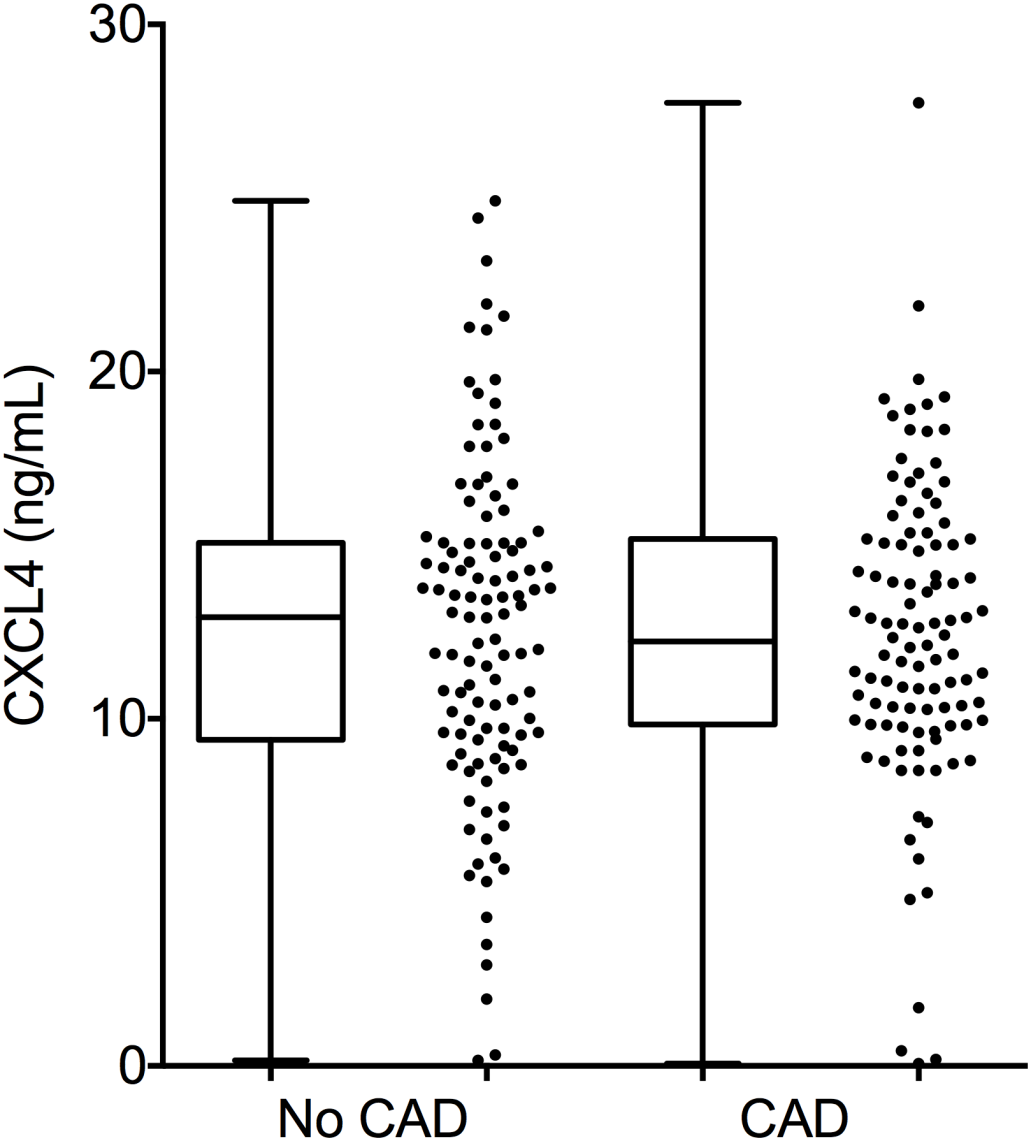
CXCL4 plasma levels in patients with or without CAD. CXCL4 plasma levels as measured by ELISA in 217 individuals undergoing coronary computed-tomography angiography (CCTA) divided by „no CAD”(n = 107) and „CAD”(n = 110). For each group, box whisker plots and dot plots are shown.

CXCL4 plasma levels did not differ between patients with or without coronary artery disease (CAD: 12.5 ± 4.5 ng/ml, no CAD: 12.5 ± 4.8 ng/ml; Fig 1). In addition, when looking at specific features such as plaque volume, total calcium score, degree of stenosis, or vascular remodeling, no association was seen with CXCL4 levels (Table 2).

**Table 2:** Plaque characteristics. Plaque characteristics of all patients and divided by CXCL4 quartiles. For each parameter the coefficient and P value with CXCL4 plasma levels is indicated as calculated by Pearson’s correlation. Continuous variables are indicated as mean ± standard deviation, categorical variables are indicated as absolute number (percentage).

Parameter	All patients (n = 217)	1^st^ quartile (n = 50)	2^nd^ quartile (n = 57)	3^rd^ quartile (n = 55)	4^th^ quartile (n = 57)	Correlation coefficient with CXCL4 levels	P value
Coronary artery disease	110 (50.7)	22 (44.0)	33 (57.9)	27 (49.1)	28 (50.0)	-0.007	0.923
Plaque volume	10.0 ± 19.7	17.7 ± 25.7	9.3 ± 17.4	7.4 ± 16.8	6.9 ± 18.5	-0.117	0.230
Total calcium score	128.3 ± 203.2	111.3 ± 171.0	168.7 ± 219.7	78.8 ± 140.2	153.5 ± 257.8	0.027	0.707
- Main stem	15.7 ± 45.8	18.9 ± 44.6	22.8 ± 62.6	7.7 ± 26.4	14.8 ± 42.1	-0.076	0.374
- LAD	79.0 ± 106.3	63.8 ± 74.8	100.7 ± 119.5	69.1 ± 114.1	78.6 ± 103.1	0.011	0.902
- LCX	23.6 ± 56.2	25.2 ± 45.4	24.3 ± 50.9	20.2 ± 68.7	25.4 ± 57.7	0.046	0.591
- RCA	44.9 ± 90.3	61.9 ± 112.1	47.0 ± 74.3	35.2 ± 91.3	38.6 ± 88.4	-0.050	0.560
Mean stenosis	15.6 ± 21.3	20.8 ± 24.4	13.3 ± 18.3	16.6 ± 23.7	12.1 ± 18.7	-0.020	0.841
Maximum stenosis	28.9 ± 27.9	31.1 ± 29.8	24.8 ± 26.1	29.6 ± 29.3	22.5 ± 26.9	-0.036	0.671
Vascular remodeling	37 (17.1)	8 (16.0)	10 (17.5)	8 (14.5)	11 (20.0)	0.036	0.654

In a logistic regression analysis taking into account both the number of cardiovascular risk factors and CXCL4 levels, CXCL4 plasma levels were not associated with the diagnosis of CAD (HR 1.017, 95%-confidence interval 0.955–1.083, P = 0.596), whereas there was a significant association between the number of cardiovascular risk factors and CAD (HR 1.691, 95%-confidence interval 1.360–2.102, P<0.001). Furthermore, there was a trend for an association between increased Agatston score (HR 1.35, 95%-confidence interval 0.99–1.84, P = 0.058) and presence of vascular remodeling (HR 1.30, 95%-confidence interval 0.964–1.753, P = 0.086) with the number of risk factors. In neither analysis, CXCL4 levels were associated with Agatston score, plaque volume, or presence of vascular remodeling.

### CXCL4 levels in patients with confirmed coronary artery disease

To test whether CXCL4 plasma levels may represent a diagnostic tool in patients with confirmed CAD, these patients were analyzed separately. Demographic and clinical data are shown in Table 3. As expected, CAD patients were older (65.5 ± 9.6 years) and more likely to be male (70 (63.6%)). In the same line, they displayed a greater number of cardiovascular risk factors (2.8 ± 1.2). As shown for the entire cohort, CXCL4 plasma levels did not correlate with any single demographic or clinical parameter, however, there was a significant inverse correlation with the total number of cardiovascular risk factors (r = -0.236, P = 0.031).

**Table 3:** Characteristics of patients with confirmed CAD. Demographic and clinical data of all patients with confirmed CAD and divided by CXCL4 quartiles. For each parameter the coefficient and P value with CXCL4 plasma levels is indicated as calculated by Pearson’s correlation. Continuous variables are indicated as mean ± standard deviation, categorical variables are indicated as absolute number (percentage).

Parameter	All patients (n = 217)	1^st^ quartile (n = 50)	2^nd^ quartile (n = 57)	3^rd^ quartile (n = 55)	4^th^ quartile (n = 57)	Correlation coefficient with CXCL4 levels	P value
**Demographics**							
Age (years)	65.5 ± 9.6	69.6 ± 8.4	63.9 ± 9.7	64.8 ± 9.8	64.7 ± 9.6	-0.129	0.186
Male sex	70 (63.6)	16 (72.2)	20 (60.6)	15 (55.6)	19 (67.9)	0.040	0.677
**Coronary risk factors**							
1. Arterial hypertension	91 (82.7)	29 (90.9)	28 (84.8)	22 (81.5)	21 (25.0)	-0.057	0.551
2. Hypercholesterolemia	78 (70.9)	18 (81.8)	24 (72.7)	20 (74.5)	16 (57.1)	-0.124	0.196
3. Diabetes mellitus	9 (8.2)	2 (9.1)	3 (9.1)	1 (3.7)	3 (10.7)	-0.038	0.697
4. Family history of coronary artery disease	54 (49.1)	13 (59.1)	15 (45.5)	14 (51.9)	12 (42.9)	-0.130	0.185
5. Smoking	37 (33.6)	7 (31.8)	15 (45.5)	7 (25.9)	8 (28.6)	-0.116	0.228
6. Obesity	17 (15.5)	6 (27.3)	6 (18.2)	2 (7.4)	3 (10.7)	-0.093	0.342
Total number of risk factors (0–5)	2.8 ± 1.2	3.2 ± 1.3	3.0 ± 1.1	2.6 ±1.0	2.4 ± 1.3	-0.236	0.031*
**Cardiac medications**							
Aspirin (100 mg/day) or clopidogrel (75 mg/day)	61 (55.5)	13 (40.9)	21 (63.6)	14 (51.9)	13 (46.4)	-0.024	0.808
Coumadin	7 (6.4)	4 (18.2)	0 (0.0)	0 (0.0)	3 (10.7)	0.005	0.957
β-blocker	61 (55.5)	18 (81.8)	18 (54.5)	12 (44.4)	13 (46.4)	-0.103	0.302
ACE inhibitor or angiotensin receptor blocker	53 (48.2)	14 (63.6)	15 (45.5)	14 (51.9)	10 (35.7)	-0.093	0.356
Statin	57 (51.8)	14 (63.6)	21 (63.6)	11 (40.7)	11 (39.3)	-0.070	0.487
Nitrates	4 (3.6)	1 (4.5)	1 (3.0)	0 (0)	2 (7.1)	0.206	0.072
**Laboratory data**							
CXCL4 (μg/mL)	12.5 ± 4.5	6.6 ± 3.2	10.8 ± 0.8	13.7 ± 0.9	17.9 ± 2.7	-	-
High sensitivity CRP (mg/L)	4 ± 7	2 ± 0	7 ± 11	2 ± 0	2 ± 1	-0.85	0.816
High sensitivity troponin T (pg/mL)	7 ± 9	14 ± 16	4 ± 2	8 ± 9	4 ± 2	-0.183	0.360

* P<0.05.

Again, CXCL4 levels were not indicative of the extemt of coronary artery disease as detected by CCTA (Table 4). Also a logistic regression analysis taking into account both the number of cardiovascular risk factors and CXCL4 levels showed that neither the number of cardiovascular risk factors nor CXCL4 levels were associated with vascular remodeling or Agatston score.

**Table 4:** Plaque characteristics of patients with confirmed CAD. Plaque characteristics of all patients with confirmed CAD and divided by CXCL4 quartiles. For each parameter the coefficient and P value with CXCL4 plasma levels is indicated as calculated by Pearson’s correlation. Continuous variables are indicated as mean ± standard deviation, categorical variables are indicated as absolute number (percentage).

Parameter	All patients (n = 217)	1^st^ quartile (n = 50)	2^nd^ quartile (n = 57)	3^rd^ quartile (n = 55)	4^th^ quartile (n = 57)	Correlation coefficient with CXCL4 levels	P value
Plaque volume	17.40 ± 24.1	17.7 ± 25.7	17.3 ± 20.8	15.9 ± 21.9	8.5 ± 22.4	-0.177	0.187
Total calcium score	199.4 ± 199.8	215.9 ± 198.3	234.4 ± 199.0	142.1 ± 170.9	199.8. ± 227.0	-0.039	0.693
- Main stem	19.5 ± 50.5	26.4 ± 51.2	25.1 ± 67.0	6.1 ± 16.4	20.0 ± 48.6	-0.084	0.417
- LAD	110.1 ± 112.6	90.0 ± 75.0	129.9 ± 124.1	108.1 ± 130.3	104.9 ± 108.3	0.018	0.860
- LCX	31.8 ± 63.6	35.3 ± 50.6	27.9 ± 52.3	32.4 ± 85.5	33.3 ± 65.0	0.064	0.538
- RCA	63.9 ± 102.6	86.6 ± 125.0	63.2 ± 81.4	56.6 ± 111.2	52.9 ± 100.4	-0.064	0.538
Mean stenosis	28.8 ± 21.9	38.5 ± 21.2	26.2 ± 18.9	28.0 ± 26.3	22.0 ± 22.0	-0.042	0.788
Maximum stenosis	43.2 ± 24.9	50.9 ± 23.4	41.3 ± 22.5	44.5 ± 27.9	36.4 ± 26.5	-0.072	0.550
Vascular remodeling	34 (65.5)	8 (36.4)	10 (30.3)	7 (25.9)	9 (32.1)	-0.017	0.864

## Discussion

A role for the platelet chemokine CXCL4 in atherosclerosis has been demonstrated in various experimental and preclinical studies with the earliest reports dating back to 1975 [24]. Over the past forty years, numerous papers have demonstrated the important role of CXCL4 in atherogenesis in murine models [9], but also in human atherosclerotic plaques [13]. A multitude of potential pro-atherogenic effects is associated with CXCL4, including promotion of monocyte adhesion to the endothelium [10], prevention of monocyte apoptosis [25], and induction of a specific macrophage phenotype termed ‘M4’ macrophage [11]. Recently, we could demonstrate that the prevalence of M4 macrophages in human coronary arteries is associated with features of plaque instability [15,26].

As mentioned before, the data on CXCL4 plasma levels and coronary artery disease are somewhat contradictory: While in many cases coronary artery disease was found to be associated with increased plasma levels [27], others did not detect differences [17,27]. One has to keep in mind that these studies were in general performed on fairly small sample sizes ranging from 46 [28] to 129 [17]. Furthermore, many of them did not only test for increased CXCL4 levels in patients with established CAD, but they also investigated subgroups with acute or recent myocardial infarction [16], patients undergoing exercise testing [29], or patients suffering from depression after myocardial infarction [28]. In some cases, heparin-releasability of CXCL4 was analyzed, i.e. CXCL4 levels were measured after intravenous administration of heparin [30].

Based on the body of data indicating an important role for CXCL4 during atherogenesis, we hypothesized that CXCL4 plasma levels may be associated with specific features of coronary artery disease such as plaque volume, calcium score, degree of stenosis, or vascular remodeling. Employing coronary computed-tomography angiography seemed an excellent way to characterize coronary artery plaques in a cohort of 217 patients. In agreement with several previous studies, we also found that CXCL4 plasma levels did not differ between patients with and with coronary artery disease. Surprisingly, there also was no association between CXCL4 levels and plaque characteristics including plaque volume, calcium score, or vascular remodeling.

To our knowledge, this study is not only the largest study investigating the potential role of CXCL4 plasma levels in CAD. Also, this is the first study to test whether CXCL4 levels may be associated with quantitative (plaque volume, calcium score) and qualitative (vascular remodeling) features of coronary atherosclerosis.

There are several explanations for our negative findings: Firstly, pre-analytical factors should be considered. It is known that application of heparin before or during blood withdrawal may affect CXCL4 levels [31]. However, none of our patients received heparin before or during blood sampling. Similarly, platelet activation may significantly affect CXCL4 levels [32]. This explanation is rather unlikely in our study because blood was drawn with extreme caution using butterfly needles with minimal venostasis, also blood samples directly underwent centrifugation in order to prevent CXCL4 release due to platelet activation. This is also supported by the fact that CXCL4 levels in our cohort were within the range of previous reports [16,17]. The fact that a substantial number of our patients was treated with aspirin or clopidogrel should not have affected CXCL4 levels [33].

Secondly, statistical issues may be underlying our negative findings. However, considering the published data on CXCL4 levels in healthy individuals ranging between 4.3 ng/mL [17] and 8.7 ng/mL [16] and in CAD patients ranging between 5.8 ng/mL and 16.0 ng/mL [16], our study was statistically powered to detect a small to medium effect size [23]. Even though, we cannot fully exclude, that there may be smaller differences of CXCL4 levels between patients with and without CAD, however, such small differences may not be relevant in clinical practice.

Thirdly, while CXCL4 may be present and active within the arterial wall, local increase of CXCL4 may not necessarily translate into systemically elevated CXCL4 levels. Platelets, which are the major source of CXCL4, may release the chemokine to the blood in micromolar concentrations [8]. Plasma levels of CXCL4 in our study range between 0 and 27 ng/ml, which is much lower than micromolar concentrations: Considering that 1 μmol/L corresponds to 7.8 μg/ml (the molecular weight of CXCL4 is 7.8 kDa), we measure levels more than three orders of magnitude below what may locally be expected. Thus, the most likely explanation for our negative findings is that measuring CXCL4 in the circulation does not reflect CXCL4 concentrations within the plaque microenvironment and therefore are not suitable to detect local plaque development and progression.

The pro-atherogenic actions associated with CXCL4 have been discussed as potential diagnostic and therapeutic targets in atherosclerosis [15,34]. The fact that measuring systemic CXCL4 levels does not seem to be a feasible approach to diagnose or quantify CAD does not generally preclude CXCL4 from being a suitable therapeutic target. Further studies are now warranted, investigating the ability of molecular imaging techniques for the local detection of CXCL4 in experimental and human atherogenesis. Similarly, blocking the pro-atherogenic functions of CXCL4 may still be an interesting approach to prevent plaque development or to induce plaque stabilization.

## Supporting Information

S1 Table Raw data. Raw data underlying the analyses.
(PDF)

## References

[1] Go AS, Mozaffarian D, Roger VL, Benjamin EJ, Berry JD, Blaha MJ, et al Heart disease and stroke statistics—2014 update: a report from the American Heart Association. Circulation 2014;129: e28–e292. 10.1161/01.cir.0000441139.02102.80 24352519 PMC5408159

[2] Huo Y, Schober A, Forlow SB, Smith DF, Hyman MC, Jung S, et al Circulating activated platelets exacerbate atherosclerosis in mice deficient in apolipoprotein E. Nat Med 2003;9: 61–67. 12483207 10.1038/nm810

[3] Hansson GK. Inflammatory mechanisms in atherosclerosis. J Thromb Haemost 2009;7 Suppl 1: 328–331. 10.1111/j.1538-7836.2009.03416.x 19630827

[4] Galkina E, Ley K. Immune and inflammatory mechanisms of atherosclerosis (*). Annu Rev Immunol 2009;27: 165–197. 10.1146/annurev.immunol.021908.132620 19302038 PMC2734407

[5] Weber C, Noels H. Atherosclerosis: current pathogenesis and therapeutic options. Nat Med 2011;17: 1410–1422. 10.1038/nm.2538 22064431

[6] Gleissner CA. Platelet-derived chemokines in atherogenesis: What’s new? Curr Vasc Pharmacol 2012; In press. 10.2174/157016112801784521 22338571

[7] Gleissner CA, von Hundelshausen P, Ley K. Platelet Chemokines in Vascular Disease. Arterioscler Thromb Vasc Biol 2008. 10.1161/ATVBAHA.108.169417 PMC2657037 18723831

[8] Brandt E, Petersen F, Ludwig A, Ehlert JE, Bock L, Flad HD. The beta-thromboglobulins and platelet factor 4: blood platelet-derived CXC chemokines with divergent roles in early neutrophil regulation. J Leukoc Biol 2000;67: 471–478. 10770278 10.1002/jlb.67.4.471

[9] Sachais BS, Turrentine T, Dawicki McKenna JM, Rux AH, Rader D, Kowalska MA. Elimination of platelet factor 4 (PF4) from platelets reduces atherosclerosis in C57Bl/6 and apoE-/- mice. Thromb Haemost 2007;98: 1108–1113. 18000617

[10] von Hundelshausen P, Koenen RR, Sack M, Mause SF, Adriaens W, Proudfoot AE, et al Heterophilic interactions of platelet factor 4 and RANTES promote monocyte arrest on endothelium. Blood 2005;105: 924–930. 15459010 10.1182/blood-2004-06-2475

[11] Gleissner CA, Shaked I, Little KM, Ley K. CXC chemokine ligand 4 induces a unique transcriptome in monocyte-derived macrophages. J Immunol 2010;184: 4810–4818. 10.4049/jimmunol.0901368 20335529 PMC3418140

[12] Gleissner CA, Shaked I, Erbel C, Bockler D, Katus HA, Ley K. CXCL4 downregulates the atheroprotective hemoglobin receptor CD163 in human macrophages. Circ Res 2010;106: 203–211. 10.1161/CIRCRESAHA.109.199505 19910578 PMC2876722

[13] Pitsilos S, Hunt J, Mohler ER, Prabhakar AM, Poncz M, Dawicki J, et al Platelet factor 4 localization in carotid atherosclerotic plaques: correlation with clinical parameters. Thromb Haemost 2003;90: 1112–1120. 14652645 10.1160/TH03-02-0069

[14] Erbel C, Tyka M, Helmes CM, Akhavanpoor M, Rupp G, Domschke G, et al CXCL4-induced plaque macrophages can be specifically identified by co-expression of MMP7+S100A8+ in vitro and in vivo. Innate Immun 2014. 10.1177/1753425914526461 24663337

[15] Erbel C, Wolf A, Lasitschka F, Linden F, Domschke G, Akhavanpoor M, et al Prevalence of M4 macrophages within human coronary atherosclerotic plaques is associated with features of plaque instability Int J Cardiol 2015; In press. 10.1016/j.ijcard.2015.03.151 25828120

[16] Levine SP, Lindenfeld J, Ellis JB, Raymond NM, Krentz LS. Increased plasma concentrations of platelet factor 4 in coronary artery disease: a measure of in vivo platelet activation and secretion. Circulation 1981;64: 626–632. 6973419 10.1161/01.cir.64.3.626

[17] De Caterina R, Gazzetti P, Mazzone A, Marzilli M, L'Abbate A. Platelet activation in angina at rest. Evidence by paired measurement of plasma beta-thromboglobulin and platelet factor 4. Eur Heart J 1988;9: 913–922. 2972544 10.1093/oxfordjournals.eurheartj.a062587

[18] Akhavanpoor M, Wangler S, Gleissner CA, Korosoglou G, Katus HA, Erbel C. Adventitial inflammation and its interaction with intimal atherosclerotic lesions. Front Physiol 2014;5: 296 10.3389/fphys.2014.00296 25152736 PMC4126462

[19] Whelton PK. Epidemiology of hypertension. Lancet 1994;344: 101–106. 7912348 10.1016/s0140-6736(94)91285-8

[20] Grundy SM, Cleeman JI, Merz CN, Brewer HB Jr, Clark LT, Hunninghake DB, et al Implications of recent clinical trials for the National Cholesterol Education Program Adult Treatment Panel III guidelines. Circulation 2004;110: 227–239. 15249516 10.1161/01.CIR.0000133317.49796.0E

[21] Korosoglou G, Lehrke S, Mueller D, Hosch W, Kauczor HU, Humpert PM, et al Determinants of troponin release in patients with stable coronary artery disease: insights from CT angiography characteristics of atherosclerotic plaque. Heart 2011;97: 823–831. 10.1136/hrt.2010.193201 20884786

[22] Gleissner CA, Erbel C, Haeussler J, Akhavanpoor M, Domschke G, Linden F, et al Low levels of natural IgM antibodies against phosphorylcholine are independently associated with vascular remodeling in patients with coronary artery disease. Clin Res Cardiol 2015;104: 13–22. 10.1007/s00392-014-0750-y 25103819

[23] Cohen J. A power primer. Psychol Bull 1992;112: 155–159. 19565683 10.1037//0033-2909.112.1.155

[24] Fuster V, Kottke BA, Ruiz CE, Lewis JC, Bowie EJ, Owen CA Jr. Proceedings: Studies on platelet factor 4 like activity in atherosclerosis susceptible and resistant pigeons. Thromb Diath Haemorrh 1975;34: 601. 1198522

[25] Scheuerer B, Ernst M, Durrbaum-Landmann I, Fleischer J, Grage-Griebenow E, Brandt E, et al The CXC-chemokine platelet factor 4 promotes monocyte survival and induces monocyte differentiation into macrophages. Blood 2000;95: 1158–1166. 10666185

[26] Erbel C, Tyka M, Helmes CM, Akhavanpoor M, Rupp G, Domschke G, et al CXCL4-induced plaque macrophages can be specifically identified by co-expression of MMP7+S100A8+ in vitro and in vivo. Innate Immun 2015;21: 255–265. 10.1177/1753425914526461 24663337

[27] Nichols AB, Owen J, Kaplan KL, Sciacca RR, Cannon PJ, Nossel HL. Fibrinopeptide A, platelet factor 4, and beta-thromboglobulin levels in coronary heart disease. Blood 1982;60: 650–654. 6179552

[28] Laghrissi-Thode F, Wagner WR, Pollock BG, Johnson PC, Finkel MS. Elevated platelet factor 4 and beta-thromboglobulin plasma levels in depressed patients with ischemic heart disease. Biol Psychiatry 1997;42: 290–295. 9270907 10.1016/S0006-3223(96)00345-9

[29] Strauss WE, Cella G, Parisi AF, Sasahara AA. Serial studies of platelet factor 4 and beta thromboglobulin during exercise in patients with coronary artery disease. Am Heart J 1985;110: 293–299. 2411121 10.1016/0002-8703(85)90147-4

[30] Abe S, Maruyama I, Arima S, Yamaguchi H, Okino H, Hamasaki S, et al Increased heparin-releasable platelet factor 4 and D dimer in patients one month after the onset of acute myocardial infarction: persistent activation of platelets and the coagulation/fibrinolytic system. Int J Cardiol 1994;47: S7–12. 7737755 10.1016/0167-5273(94)90320-4

[31] Sadayasu T, Nakashima Y, Yashiro A, Kawashima T, Kuroiwa A. Heparin-releasable platelet factor 4 in patients with coronary artery disease. Clin Cardiol 1991;14: 725–729. 1835913 10.1002/clc.4960140906

[32] Levine SP, Krentz LS. Development of a radioimmunoassay for human platelet factor 4. Thromb Res 1977;11: 673–686. 412272 10.1016/0049-3848(77)90025-1

[33] Cella G, Colby SI, Taylor AD, McCracken L, Parisi AF, Sasahara AA. Platelet factor 4 (PF4) and heparin-released platelet factor 4 (HR-PF4) in patients with cardiovascular disorders. Thromb Res 1983;29: 499–509. 6222505 10.1016/0049-3848(83)90345-6

[34] Koenen RR, von Hundelshausen P, Nesmelova IV, Zernecke A, Liehn EA, Sarabi A, et al Disrupting functional interactions between platelet chemokines inhibits atherosclerosis in hyperlipidemic mice. Nat Med 2009;15: 97–103. 10.1038/nm.1898 19122657

