# Supplementary material for: CXCL4 Plasma Levels Are Not Associated with the Extent of Coronary Artery Disease or with Coronary Plaque Morphology
Source: PLoS One. 2015 Nov 2;10(11):e0141693. doi: 10.1371/journal.pone.0141693 (PMC4629911; doi:10.1371/journal.pone.0141693)
Supplement: S1 Table — Raw data underlying the analyses. (PDF) [file pone.0141693.s001.pdf]

Pat#	CXCL4	hsTnT	hsCRT	Agatston	Plaquevolume	Remodeling	Mainstem	LAD	LCX	RCA	Alter	Gender	Hypertension	Cholesterol	Diabetes	Familyhistory	Tobacco	Adipositas	#Riskfactors	CAD	meanstenosis	maxstenosis	ASS-Clopi	Anticoagulant	Statin	ß-Blocker	ACE-AT	Nitrate
1	0,19	3,64	2,00	98	4,60	0	.	.	.	.	79,32	1	0	0	0	1	0	0	1	1	0,00	0,00	1	0	0	1	.	.
2	5,82	.	.	.	.	.	.	.	.	.	.	.	.	.	.	.	.	.	0	.	.	.	.	.	.	.	.	.
3	13,31	10,23	0,61	31	0,00	0	0	31	0	0	69,43	0	1	1	0	1	0	0	3	1	.	.	1	0	0	0	1	0
4	13,54	.	.	0	.	.	.	.	.	.	47,34	0	.	.	.	.	.	.	0	.	.	.	.	.	.	.	.	.
5	13,56	.	.	0	0,00	0	.	.	.	.	.	1	0	0	0	1	1	0	2	0	.	.	.	.	.	.	.	.
6	10,20	3,00	9,40	0	.	.	.	.	.	.	68,66	1	1	.	.	.	0	.	1	0	0,00	0,00	0	0	0	0	0	.
7	10,68	7,59	0,76	143	27,50	0	17	121	5	0	59,11	0	1	1	0	0	1	0	3	1	.	.	.	.	.	.	.	.
8	19,09	9,00	2,40	20,3	.	.	.	.	.	.	70,21	0	1	1	.	.	.	.	2	0	31,00	31,00	0	0	0	0	0	.
9	16,49	10,46	1,14	3,4	0,00	0	0	3	0	0	45,48	1	0	0	0	0	0	0	0	1	0,00	0,00	0	0	0	0	0	.
10	11,83	11,74	0,47	41	5,70	0	0	41	0	0	46,28	1	1	1	0	0	1	0	3	0	.	.	.	.	.	.	.	.
11	12,91	3,00	3,70	0	.	.	.	.	.	.	61,55	0	1	1	.	.	.	.	2	0	0,00	0,00	0	0	0	0	0	.
12	18,48	.	.	0	.	.	.	.	.	.	65,91	0	.	.	.	.	.	.	0	.	.	.	.	.	.	.	.	.
13	12,93	9,08	1,65	191	0,00	0	143	48	0	0	68,42	0	1	1	0	0	0	0	2	0	47,50	62,00	1	0	1	1	0	0
14	16,83	11,40	2,51	68	0,00	0	0	68	0	0	61,74	1	0	0	0	1	1	0	2	1	.	.	.	.	.	.	.	.
15	14,67	.	.	0	.	.	.	.	.	.	59,31	0	.	.	.	.	.	.	0	.	.	.	.	.	.	.	.	.
16	15,83	40,00	2,00	381	.	.	.	.	.	.	58,20	0	1	1	.	.	1	.	3	0	57,60	73,00	1	0	1	1	1	.
17	14,24	27,00	1,41	432	4,30	0	0	187	25	221	68,33	1	1	1	0	0	0	0	2	1	48,83	60,00	0	0	0	0	0	.
18	15,64	7,00	2,05	64	18,00	0	0	64	0	0	68,69	1	0	0	0	0	0	0	0	1	34,33	52,00	0	0	0	0	0	0
19	6,53	3,00	2,00	0	.	.	.	.	.	.	62,41	0	.	0	.	.	0	.	0	0	0,00	0,00	0	0	0	0	.	.
20	11,66	4,00	2,70	0	.	.	.	.	.	.	64,29	0	1	1	.	.	0	.	2	0	23,00	23,00	0	0	1	1	1	.
21	7,18	13,93	1,33	130	20,00	0	10	80	29	11	82,48	1	1	0	0	0	0	1	2	1	40,00	51,00	0	1	0	1	1	0
22	8,89	12,09	4,28	241	18,00	0	135	106	0	0	77,16	0	0	1	0	1	1	1	4	1	.	.	0	1	0	0	0	0
23	6,52	41,00	3,04	92	0,60	1	0	40	14	40	69,82	1	1	1	0	1	1	0	4	1	67,00	88,00	1	0	1	1	1	0
24	21,89	12,05	2,83	298,7	22,50	1	92	135	69	3	71,15	1	1	0	0	1	0	0	2	1	.	.	1	0	0	0	0	0
25	11,09	6,36	0,93	16	0,00	0	16	0	0	0	51,10	1	1	0	0	0	1	0	2	1	48,00	48,00	0	0	1	0	0	.
26	14,05	3,00	2,00	.	.	.	.	.	.	.	51,76	0	.	1	.	.	1	.	2	0	0,00	0,00	0	0	0	0	0	.
27	24,92	15,00	54,70	83	.	.	.	.	.	.	64,29	0	.	.	.	.	.	.	0	.	.	.	.	.	.	.	.	.
28	19,27	6,00	0,80	178	6,60	0	0	134	38	6	71,13	1	0	0	0	0	0	0	0	1	43,50	46,00	0	0	0	0	0	.
29	12,62	12,27	5,45	44	0,00	0	0	6	38	0	78,51	0	1	0	0	0	0	0	1	1	.	.	1	0	0	1	1	0
30	17,85	.	.	.	.	.	.	.	.	.	.	0	.	.	.	.	.	.	0	.	.	.	.	.	.	.	.	.
31	15,40	4,12	4,82	0	0,00	0	0	0	.	0	48,06	0	1	0	0	0	0	0	1	0	0,00	0,00	0	0	0	0	1	0
32	10,98	7,56	1,06	0	0,00	0	0	0	0	0	59,09	0	0	1	0	0	0	0	1	0	0,00	0,00	0	0	0	0	0	0
33	21,28	9,89	0,54	17	15,00	1	0	0	15	2	70,90	0	1	1	0	1	0	0	3	0	0,00	0,00	1	0	1	1	1	0
34	0,32	4,91	4,09	54	25,00	0	0	0	0	0	55,94	0	1	1	0	1	1	1	5	0	0,00	0,00	1	0	1	1	1	0
35	15,00	10,47	0,99	22	7,50	0	0	8	0	14	55,51	1	1	0	0	1	0	0	2	1	.	.	1	0	1	1	0	0
36	13,72	6,37	0,91	0	0,00	0	0	0	0	0	51,77	0	1	1	0	1	0	0	3	0	0,00	0,00	1	0	0	1	0	0
37	11,52	9,36	0,64	0	0,00	0	0	0	0	0	50,63	1	0	0	0	0	0	0	0	0	0,00	0,00	0	0	0	0	0	0
38	8,71	34,36	5,93	469	79,80	1	0	304	164	1	74,94	0	1	1	0	1	0	1	4	1	42,50	46,00	1	0	0	1	1	1
39	10,76	.	.	7	.	.	.	.	.	.	86,46	0	.	.	.	.	.	.	0	.	.	.	.	.	.	.	.	.
40	10,31	5,45	2,85	390	.	1	0	161	0	229	68,72	1	1	1	0	0	1	0	3	1	41,17	47,00	1	0	1	1	1	0
41	9,97	4,05	3,89	158	15,50	0	0	72	7	79	49,86	1	1	0	0	1	1	0	3	1	45,33	50,00	1	0	0	0	0	0
42	9,42	15,46	0,51	0	0,00	0	0	19	18	1	60,79	1	1	1	0	0	0	1	3	1	39,00	39,00	1	0	1	1	1	1
43	16,42	10,26	15,50	5,7	0,00	0	0	6	0	0	54,31	0	1	1	1	1	0	1	5	0	0,00	0,00	1	0	0	1	1	0
44	10,87	28,20	2,45	315	67,80	1	0	289	1	25	82,77	1	1	1	0	0	1	0	3	1	.	.	1	0	1	1	1	1
45	13,87	7,70	1,02	22	0,00	0	0	0	22	0	55,74	1	1	0	0	1	1	0	3	1	.	.	.	.	.	.	.	.
46	9,84	3,00	2,00	36	.	.	.	.	.	.	76,19	1	0	1	.	.	0	.	1	1	0,00	0,00	1	0	1	0	0	.
47	21,95	3,00	6,50	11	.	.	.	.	.	.	56,53	1	1	1	0	.	0	.	2	0	0,00	0,00	1	0	1	1	1	.
48	4,28	3,00	4,70	0	.	.	.	.	.	.	71,78	0	1	0	0	.	0	.	1	0	0,00	0,00	0	0	0	1	.	.
49	15,01	10,33	1,54	117	0,00	0	0	93	23	1	49,06	1	1	1	0	1	0	0	3	1	48,67	59,00	0	0	0	1	1	0
50	10,01	9,47	2,23	0	0,00	0	0	0	0	0	74,62	0	1	1	0	0	0	0	2	0	0,00	0,00	1	0	1	1	0	0
51	14,52	3,00	2,00	0	0,00	0	.	.	.	.	57,04	0	0	0	0	1	1	0	2	0	0,00	0,00	0	0	0	0	0	.
52	19,77	21,00	2,00	0	.	.	.	.	.	.	72,76	0	.	.	.	.	.	.	0	.	.	.	.	.	.	.	.	.
53	11,12	12,57	1,01	53	10,20	1	5	12	0	36	69,99	1	1	1	0	0	1	0	3	1	32,00	42,00	1	0	1	1	0	0
54	9,96	3,00	0,49	365	.	0	83	54	25	203	73,12	1	1	1	0	0	0	0	2	1	46,60	66,00	0	0	1	1	0	0
55	16,75	3,00	2,00	0	.	.	.	.	.	.	70,13	0	0	0	0	.	0	.	0	0	0,00	0,00	0	0	0	0	0	.
56	11,51	7,85	23,00	128	0,00	0	.	.	.	.	67,33	1	0	1	0	0	0	0	1	1	32,00	32,00	1	0	1	1	0	.
57	19,22	12,85	5,23	35	23,60	0	.	.	.	.	62,30	1	1	1	0	0	1	1	4	1	.	.	.	.	.	.	.	.
58	9,83	16,01	0,42	378	32,00	1	0	346	0	32	61,13	1	1	0	0	1	1	0	3	1	0,00	0,00	1	0	0	0	1	0
59	9,09	3,00	2,00	6	0,00	0	.	.	.	.	46,24	0	1	0	0	1	0	0	2	0	0,00	0,00	0	0	0	0	0	.
60	12,05	6,00	2,34	275	.	1	0	162	66	47	62,01	1	1	1	0	0	1	0	3	1	32,33	57,00	0	0	0	0	0	.
61	15,04	8,03	1,29	0	0,00	0	0	0	0	0	52,64	0	0	0	0	1	1	0	2	0	0,00	0,00	0	0	0	0	1	0
62	15,24	.	.	891	.	.	.	.	.	.	75,77	1	.	.	.	.	.	.	0	.	.	.	.	.	.	.	.	.
63	10,87	.	2,00	56	0,50	0	.	.	.	.	71,27	0	1	1	0	0	1	0	3	1	33,00	33,00	1	0	0	0	1	.
64	19,70	.	2,00	0	.	.	.	.	.	.	68,23	0	1	1	0	.	1	.	3	0	0,00	0,00	0	0	0	0	0	.
65	14,10	3,00	0,49	225	12,00	0	0	199	21	5	67,35	1	1	0	0	0	1	0	2	1	0,00	0,00	0	0	0	0	0	.
66	15,35	.	.	124	41,25	0	.	.	.	.	.	0	1	1	0	1	0	0	3	1	.	.	.	.	.	.	.	.
67	24,42	.	.	.	.	.	.	.	.	.	.	1	.	.	.	.	.	.	0	.	.	.	.	.	.	.	.	.
68	13,97	.	.	.	.	.	.	.	.	.	.	1	.	.	.	.	.	.	0	.	.	.	.	.	.	.	.	.
69	8,57	18,69	0,68	2	0,00	0	0	0	0	0	68,25	0	0	0	0	0	1	0	1	0	.	.	0	0	0	1	0	0
70	10,77	.	.	970	.	.	.	.	.	.	67,28	1	.	.	.	.	.	.	0	.	.	.	.	.	.	.	.	.
71	18,08	19,00	.	4	.	.	.	.	.	.	73,43	0	.	.	.	.	.	.	0	.	.	.	.	.	.	.	.	.
72	10,44	3,00	0,32	282	.	0	0	125	12	143	69,28	0	1	1	0	1	0	1	4	1	.	.	1	0	1	1	1	0
73	13,43	7,43	0,65	0	0,00	0	0	0	0	0	64,40	0	1	0	1	0	0	0	2	0	.	.	.	.	.	.	.	.
74	10,48	6,00	4,50	227	.	.	.	.	.	.	87,14	1	.	.	.	.	.	.	0	.	.	.	.	.	.	.	.	.
75	19,06	3,00	2,52	321	0,00	1	0	52	204	65	64,15	1	1	1	0	1	0	0	3	1	38,00	41,00	1	1	1	1	0	0
76	21,20	8,26	2,18	1	0,00	0	0	0	1	0	62,18	1	1	1	0	0	1	0	3	0	0,00	0,00	1	0	1	1	0	0
77	16,76	15,66	7,80	250	38,00	1	0	5	0	0	67,82	0	1	0	0	0	0	0	1	0	0,00	0,00	1	1	0	1	1	0
78	12,75	.	.	721	18,00	0	.	.	.	.	.	1	0	0	0	1	0	0	1	1	.	.	.	.	.	.	.	.
79	11,70	9,56	0,18	83	0,00	0	0	33	14	36	65,15	0	1	1	0	1	0	0	3	1	.	.	1	0	1	1	0	0

80	15,07	5,00	2,00	45	.	.	.	.	.	.	58,67	0	1	0	0	.	0	.	1	0	24,00	24,00	0	0	0	1	0	.
81	8,20	3,00	2,00	71	.	.	.	.	.	.	61,75	1	1	1	0	.	0	.	2	0	37,33	51,00	0	0	0	0	1	.
82	14,83	5,06	0,52	260	26,00	1	0	15	0	0	60,10	1	1	1	0	1	0	0	3	1	.	.	1	0	1	1	0	0
83	10,38	<3	38,20	571	.	0	0	480	54	37	60,77	0	1	1	1	1	1	1	6	1	.	.	1	0	1	0	0	0
84	17,85	9,14	2,02	0	0,00	0	0	0	0	0	55,86	1	1	0	0	0	1	0	2	0	.	.	.	.	.	.	.	.
85	11,32	20,05	6,11	126	35,00	0	0	87	9	30	62,07	0	1	0	1	0	0	1	3	1	.	.	0	0	0	1	1	0
86	15,18	16,72	13,60	460	35,50	0	0	120	0	340	56,08	0	0	1	0	0	1	0	2	1	.	.	0	1	1	0	0	0
87	5,67	8,00	2,00	0	.	.	.	.	.	.	67,67	0	1	1	0	.	0	.	2	0	60,00	60,00	1	0	1	1	.	.
88	2,91	3,00	14,80	0	.	.	.	.	.	.	64,38	0	0	0	0	.	0	.	0	0	0,00	0,00	0	0	1	0	.	.
89	8,48	13,00	2,00	0	.	.	.	.	.	.	61,10	.	0	0	0	.	0	.	0	0	0,00	0,00	0	0	0	0	0	.
90	15,07	4,57	5,70	0	.	.	.	.	.	.	71,88	1	1	1	0	.	0	.	2	0	0,00	0,00	0	0	1	1	1	.
91	14,06	14,00	2,10	416	65,00	1	56	233	20	107	82,71	1	1	1	0	0	0	0	2	1	51,00	72,00	1	0	1	0	1	0
92	11,05	7,91	3,56	356	35,00	1	0	0	0	0	41,84	0	1	0	0	1	0	1	3	1	.	.	0	0	0	0	0	0
93	7,45	.	.	0	.	.	.	.	.	.	57,30	0	.	.	.	.	.	.	0	.	.	.	.	.	.	.	.	.
94	16,01	.	.	.	.	.	.	.	.	.	.	1	.	.	.	.	.	.	0	.	.	.	.	.	.	.	.	.
95	13,76	5,51	4,21	2	0,00	1	0	0	2	0	37,98	1	0	0	0	0	1	0	1	0	.	.	1	0	0	1	0	0
96	8,68	10,65	0,37	0	0,00	0	0	0	0	0	65,52	0	1	1	0	1	0	0	3	0	0,00	0,00	0	0	1	1	1	0
97	18,47	3,00	5,00	0	.	.	.	.	.	.	62,12	0	1	0	0	.	0	.	1	0	0,00	0,00	0	0	0	0	0	.
98	7,32	3,00	2,00	49	.	.	.	.	.	.	75,81	1	1	1	0	.	0	.	2	0	0,00	0,00	0	0	1	0	.	.
99	11,17	8,52	2,07	266	16,00	0	0	266	0	0	59,84	0	1	1	0	1	0	0	3	1	49,75	63,00	1	0	1	1	0	0
100	17,07	15,01	8,04	53	0,00	0	0	0	0	53	64,43	0	1	1	0	0	0	1	3	1	43,50	54,00	1	0	1	1	1	0
101	11,88	3,00	2,00	0	.	.	.	.	.	.	73,46	0	1	0	0	.	0	.	1	0	0,00	0,00	0	0	0	0	0	.
102	5,49	3,00	2,40	260,7	.	.	.	.	.	.	67,88	0	1	1	0	.	0	.	2	0	26,00	26,00	0	1	1	1	.	.
103	5,99	3,00	2,00	0	.	.	.	.	.	.	48,27	0	0	0	0	.	0	.	0	0	0,00	0,00	0	0	0	0	.	.
104	13,51	6,00	0,47	0	0,00	0	0	0	0	0	59,71	0	0	1	0	1	0	0	2	0	0,00	0,00	0	0	1	0	1	.
105	10,47	15,93	0,98	55	25,00	1	5	4	25	0	80,51	0	1	1	0	0	0	0	2	1	36,00	36,00	1	0	0	1	1	0
106	9,53	.	.	150	.	.	.	.	.	.	68,82	1	.	.	.	.	.	.	0	.	.	.	.	.	.	.	.	.
107	15,06	19,18	1,21	36	22,00	0	0	36	0	0	56,54	1	0	1	0	1	0	0	2	1	0,00	0,00	0	0	0	0	0	.
108	16,28	.	.	43	0,00	0	.	.	.	.	.	1	1	0	0	1	0	0	2	1	.	.	.	.	.	.	.	.
109	11,89	8,76	1,06	0	0,00	0	.	.	.	.	54,88	0	1	0	0	0	0	0	1	0	0,00	0,00	0	0	0	0	1	0
110	15,01	5,29	1,28	50	.	0	39	10	1	0	69,76	1	1	1	0	0	0	0	2	1	38,00	46,00	1	0	1	1	1	0
111	4,80	6,66	3,34	156	5,10	0	0	75	35	46	57,31	1	1	0	1	1	1	0	4	1	.	.	0	0	0	1	0	0
112	10,27	3,00	0,28	117	.	0	0	150	18	9	51,29	1	0	1	0	1	1	1	4	1	43,20	64,00	1	0	1	0	0	.
113	8,80	5,00	1,08	607	40,70	1	0	191	215	201	64,21	1	1	1	0	0	0	0	2	1	42,86	61,00	1	0	0	1	1	0
114	12,75	12,07	5,20	269,1	63,00	1	0	71	0	198	73,41	0	1	1	0	0	0	0	2	1	58,20	84,00	1	0	1	1	1	0
115	13,90	3,00	2,15	62	.	0	0	40	0	22	69,99	1	1	1	0	1	0	0	3	1	41,25	47,00	1	0	0	1	1	0
116	11,36	13,57	0,68	348	35,60	0	172	124	5	47	70,56	1	1	1	0	1	0	0	3	1	42,00	50,00	0	0	1	1	1	0
117	13,06	7,79	0,70	0	0,00	0	0	0	0	0	50,77	1	1	1	0	1	0	0	3	0	.	.	0	0	0	0	0	0
118	16,96	12,68	0,91	0	0,00	0	0	0	0	0	56,38	1	1	0	0	0	0	0	1	0	0,00	0,00	0	0	0	0	1	0
119	9,73	11,13	2,89	197	0,00	0	172	19	6	0	62,88	0	1	0	0	0	1	0	2	0	0,00	0,00	1	0	0	0	1	0
120	0,44	3,00	0,54	123	20,00	1	106	17	0	0	67,91	1	1	1	0	1	0	0	3	1	.	.	1	0	1	0	0	0
121	19,78	7,52	6,24	11	0,00	1	10	1	0	0	65,74	0	1	1	0	1	0	0	3	1	.	.	1	0	1	1	1	0
122	12,17	9,21	0,94	0	0,00	0	0	0	0	0	54,55	0	1	0	0	0	1	1	3	0	0,00	0,00	0	0	0	1	1	0
123	5,96	23,86	6,30	16	27,00	1	0	0	0	16	82,51	0	1	0	0	0	0	0	1	1	42,00	42,00	0	1	0	0	1	0
124	0,16	10,71	1,41	0	0,00	0	0	0	0	0	72,88	0	1	1	0	1	0	0	3	0	.	.	0	1	0	1	0	0
125	13,09	3,25	1,88	9	0,00	0	0	9	0	0	51,33	1	1	1	0	0	1	0	3	1	.	.	0	0	0	0	0	0
126	18,91	15,22	1,32	283	32,70	1	0	225	0	58	72,30	1	1	1	0	0	0	0	2	1	29,00	29,00	1	0	1	0	1	0
127	14,10	3,00	2,00	0	.	.	.	.	.	.	65,91	0	0	0	0	.	0	.	0	0	0,00	0,00	0	0	0	0	0	.
128	14,12	11,64	.	68	52,30	0	.	.	.	.	80,11	0	1	0	1	0	0	1	3	1	69,00	69,00	0	0	0	0	0	.
129	8,51	5,62	0,66	138	0,00	0	0	128	8	2	63,02	0	1	1	0	1	0	0	3	1	38,00	59,00	1	0	1	1	0	0
130	10,32	10,30	3,41	18	0,00	0	0	18	0	0	64,14	0	1	0	0	1	0	0	2	1	0,00	0,00	0	0	0	1	1	0
131	9,63	3,00	1,04	448	29,60	0	301	.	23	125	59,70	1	1	1	0	1	1	0	4	1	39,50	51,00	1	0	1	0	1	0
132	10,55	8,57	0,17	0	0,00	0	0	0	0	0	48,26	0	0	0	0	1	0	0	1	0	37,00	37,00	0	0	0	0	0	0
133	13,76	8,88	0,33	0	0,00	0	0	0	0	0	51,54	1	1	0	0	0	1	1	3	0	.	.	1	0	1	0	0	0
134	13,88	3,00	2,00	197	.	1	0	190	3	4	57,06	1	1	1	0	1	1	0	4	1	40,50	47,00	1	0	1	1	1	0
135	14,35	9,47	6,28	0	0,00	0	0	0	0	0	60,62	1	1	0	0	1	0	0	2	0	.	.	1	0	1	1	0	0
136	9,39	.	.	.	.	.	.	.	.	.	.	0	.	.	.	.	.	.	0	.	.	.	.	.	.	.	.	.
137	9,82	7,31	0,72	512	7,10	0	128	180	116	88	68,63	0	1	1	0	0	0	0	2	1	.	.	1	0	1	1	0	0
138	15,93	3,00	1,59	75	0,00	1	0	35	5	35	57,58	1	1	1	0	0	0	0	2	1	0,00	0,00	0	0	0	0	0	.
139	8,99	8,12	1,33	0	0,00	0	0	0	0	0	65,59	0	1	0	0	0	0	0	1	0	0,00	0,00	1	0	0	1	1	0
140	9,73	7,00	2,00	.	.	.	.	.	.	.	62,18	0	1	0	0	.	0	.	1	0	0,00	0,00	0	0	0	0	1	.
141	12,29	.	.	.	.	.	.	.	.	.	73,36	0	.	.	.	.	.	.	0	.	.	.	.	.	.	.	.	.
142	13,94	7,82	0,93	14	25,00	1	0	362	356	232	74,01	0	1	1	0	0	0	0	2	1	.	.	1	0	1	1	1	0
143	9,56	.	.	3	.	.	.	.	.	.	69,53	0	.	0	.	.	.	.	0	.	.	.	.	.	.	.	.	.
144	11,13	.	3,90	41,5	.	.	.	.	.	.	51,62	1	1	0	0	.	1	.	2	0	0,00	0,00	0	0	0	0	0	.
145	6,81	3,00	2,00	0	.	.	.	.	.	.	50,89	0	1	1	0	.	0	.	2	0	0,00	0,00	0	0	1	1	.	.
146	9,61	20,00	15,60	0	0,00	0	.	.	.	.	.	0	1	0	1	0	0	0	2	0	.	.	.	.	.	.	.	.
147	9,80	11,63	0,76	851	12,00	1	0	328	210	313	70,07	1	1	1	1	0	0	0	3	1	51,83	64,00	1	0	1	0	1	0
148	15,18	10,73	3,55	116	10,40	0	0	104	12	0	62,87	1	1	1	0	1	0	0	3	1	49,00	59,00	0	0	0	1	1	0
149	18,32	9,59	0,28	75	10,20	0	20	12	7	36	69,28	0	0	0	0	1	0	0	1	1	53,00	65,00	0	0	0	0	0	.
150	16,81	9,01	5,25	628	35,70	1	132	388	46	62	68,73	1	1	1	0	0	1	1	4	1	49,60	69,00	1	0	1	1	1	0
151	8,67	3,00	2,00	0	.	.	.	.	.	.	63,79	0	1	1	0	.	0	.	2	0	0,00	0,00	0	0	1	1	0	.
152	12,41	8,32	1,60	259	7,00	0	0	259	0	0	67,29	0	1	1	0	1	0	0	3	1	50,67	65,00	1	0	1	1	1	0
153	9,76	3,00	2,00	46	.	0	0	2	0	44	63,78	1	0	1	0	1	0	0	2	1	48,00	48,00	1	0	1	0	0	0
154	16,99	3,00	2,00	51	.	.	.	.	.	.	52,22	1	1	0	1	.	0	.	2	1	51,50	54,00	0	0	0	0	0	.
155	11,83	13,23	1,80	183	66,60	1	0	127	21	35	41,38	1	1	1	0	1	0	0	3	1	48,20	71,00	1	0	1	1	0	0
156	10,34	3,00	0,83	10	0,00	0	.	.	.	.	53,14	1	0	1	0	1	0	0	2	1	0,00	0,00	0	0	0	0	0	.
157	12,73	8,09	0,45	13	10,50	0	0	13	0	0	73,77	1	1	0	0	0	0	0	1	1	.	.	0	0	0	1	0	0
158	11,85	3,00	2,00	0	.	.	.	.	.	.	61,58	0	0	0	0	.	0	.	0	.	0,00	0,00	0	0	0	0	0	.
159	13,27	13,30	2,00	24	.	.	.	.	.	.	69,44	0	0	0	0	.	0	.	0	0	42,50	43,00	0	0	0	0	0	.

160	18,73	6,77	0,57	156	0,00	0	0	90	20	46	58,81	1	0	0	0	0	1	0	1	1	.	.	1	0	0	1	0	0
161	15,05	3,00	2,00	3,6	.	.	.	.	.	.	62,67	0	1	1	1	.	0	.	3	0	45,00	45,00	1	0	1	1	1	.
162	16,21	11,71	2,74	156	0,00	0	0	151	5	0	78,25	0	1	1	0	0	0	0	2	1	0,00	0,00	1	0	1	1	0	0
163	9,96	10,99	0,80	0	0,00	0	0	0	0	0	47,04	1	0	0	0	1	1	0	2	0	.	.	0	0	0	1	0	0
164	12,83	12,47	1,31	0	7,50	1	0	0	0	0	60,95	0	1	1	0	0	0	0	2	1	25,00	25,00	0	0	0	0	1	0
165	14,84	11,86	0,64	0	0,00	0	0	0	0	0	55,61	1	0	0	0	0	0	0	0	0	0,00	0,00	0	0	0	0	0	.
166	17,37	16,91	0,61	456	85,00	1	0	140	189	66	79,41	1	1	1	1	0	0	0	3	1	.	.	1	0	1	1	0	0
167	14,27	8,04	1,51	23	0,00	0	0	23	0	0	65,00	1	1	1	0	0	0	0	2	0	38,00	38,00	0	0	1	1	0	0
168	12,34	4,09	6,85	128	20,70	1	0	127	1	0	54,49	0	0	1	0	1	1	0	3	1	.	.	.	.	.	.	.	.
169	8,71	7,36	5,10	0	0,00	0	.	.	.	.	62,16	1	0	1	0	0	0	0	1	0	0,00	0,00	1	0	0	0	0	.
170	17,50	23,28	1,99	854	47,50	1	32	234	193	395	81,82	0	1	1	1	0	1	0	4	1	.	.	1	0	0	1	1	1
171	18,28	26,60	0,50	8,5	24,00	0	0	9	0	0	58,18	1	1	0	0	1	0	0	2	1	0,00	0,00	0	0	0	0	1	0
172	12,92	6,44	1,21	120	5,70	0	0	53	2	65	51,63	0	0	1	0	1	1	0	3	1	.	.	1	0	0	0	0	0
173	15,06	3,00	2,00	0	0,00	0	.	.	.	.	63,58	0	0	1	0	0	0	0	1	0	0,00	0,00	0	0	0	0	0	.
174	8,78	23,36	21,20	136	.	.	.	.	.	.	72,08	1	1	1	0	.	0	.	2	1	25,00	29,00	1	0	1	1	1	.
175	7,01	10,14	4,59	21	11,20	0	0	19	0	2	53,25	1	1	1	0	0	0	1	0	3	1	44,00	60,00	0	0	0	0	.	.
176	13,11	3,00	2,00	36	.	.	.	.	.	.	54,42	0	1	1	0	.	0	.	2	1	0,00	0,00	1	0	1	0	1	.
177	12,12	10,87	1,92	54,06	16,00	0	0	52	0	2	61,34	1	1	0	0	0	1	0	2	1	.	.	0	0	1	1	1	0
178	10,40	7,00	3,10	0	.	.	.	.	.	.	68,14	0	1	1	0	.	1	.	3	0	0,00	0,00	1	0	1	0	2	.
179	11,64	8,00	1,03	494	71,00	0	0	271	176	47	76,24	1	1	0	0	0	1	0	2	1	35,00	45,00	0	0	0	1	1	0
180	3,50	13,26	2,13	0	0,00	0	0	0	0	0	76,17	1	1	1	1	1	0	1	5	0	.	.	1	0	0	1	0	0
181	16,77	8,87	1,42	0	0,00	0	0	0	0	0	66,61	0	1	1	0	0	1	0	3	0	0,00	0,00	0	0	0	0	0	0
182	19,37	8,48	2,00	1274	.	.	.	.	.	.	76,63	1	.	.	.	.	.	.	0	.	.	.	.	.	.	.	.	.
183	15,35	5,13	0,44	9	0,00	0	0	9	0	0	44,22	0	1	0	0	1	1	0	3	1	.	.	0	0	0	1	0	0
184	23,19	7,00	2,00	18,4	.	.	.	.	.	.	71,71	0	1	1	0	.	0	.	2	0	43,00	43,00	1	0	1	1	0	.
185	9,22	3,00	2,00	24	.	.	.	.	.	.	66,08	0	1	1	0	.	1	.	3	.	34,00	34,00	0	0	0	0	1	.
186	13,72	14,00	1,86	0	0,00	0	.	.	.	.	.	0	1	0	0	0	0	0	1	0	.	.	.	.	.	.	.	.
187	9,08	32,63	4,65	170	58,00	1	8	7	74	81	68,91	1	1	1	0	0	0	0	2	1	42,80	61,00	0	0	1	1	1	.
188	9,08	<3	3,15	286	82,00	0	114	.	63	109	70,76	1	1	1	0	1	1	0	4	1	42,80	61,00	0	0	1	1	1	.
189	9,61	3,00	10,20	0	0,00	0	.	.	.	.	73,61	1	1	1	0	0	0	0	2	0	0,00	0,00	0	0	0	1	1	.
190	14,38	3,00	2,90	0	.	.	.	.	.	.	73,93	0	0	0	0	.	1	.	1	0	0,00	0,00	0	0	0	0	0	.
191	27,74	3,00	2,00	21	.	0	0	12	9	0	53,89	1	1	1	0	0	0	0	2	1	33,00	33,00	1	0	1	1	0	1
192	13,02	3,00	8,70	0	.	.	.	.	.	.	44,99	0	1	1	1	.	0	.	3	.	57,00	62,00	1	0	0	1	1	.
193	12,00	5,74	2,00	0	0,00	0	0	0	0	0	59,00	0	1	1	0	0	0	0	2	0	0,00	0,00	0	0	0	0	0	0
194	16,26	9,33	0,96	8	0,00	0	8	0	0	0	59,71	1	1	1	0	1	1	0	4	0	0,00	0,00	1	0	1	1	1	0
195	15,85	23,05	8,23	583	87,50	1	191	360	1	31	70,34	1	1	1	0	1	1	0	4	1	49,67	69,00	1	0	1	1	2	.
196	8,85	3,03	10,60	0	0,00	0	0	0	0	0	58,51	0	1	1	0	1	0	1	4	0	0,00	0,00	0	0	1	0	0	0
197	11,86	14,91	0,69	38	0,00	0	0	19	18	1	64,28	0	1	1	0	0	0	1	3	1	.	.	1	0	1	1	1	0
198	14,28	7,19	4,10	0	0,00	0	0	0	0	0	61,19	0	0	1	0	1	0	1	3	0	0,00	0,00	0	0	0	0	0	0
199	5,31	18,00	6,05	0	0,00	0	.	.	.	.	.	1	1	1	0	0	0	1	3	0	.	.	.	.	.	.	.	.
200	12,90	8,95	0,69	56	0,00	0	0	56	0	0	71,95	0	0	1	0	0	0	0	1	1	.	.	0	0	0	0	1	0
201	10,81	9,17	0,90	0	0,00	0	0	11	0	0	58,00	1	0	0	0	1	0	0	1	0	0,00	0,00	0	0	0	0	0	.
202	6,92	5,78	2,31	16	0,00	0	0	13	0	3	58,18	1	1	0	0	0	0	0	1	0	0,00	0,00	0	0	0	0	.	.
203	1,93	.	2,00	809,29	.	.	.	.	.	.	79,18	1	.	.	.	.	.	.	0	.	.	.	.	.	.	.	.	.
204	13,65	3,00	0,94	284	0,00	0	.	.	.	.	.	1	1	1	0	1	1	0	4	1	41,00	49,00	1	0	1	1	1	.
205	1,68	.	4,59	0	0,00	0	0	117	0	0	64,90	1	1	1	0	0	1	0	3	1	.	.	1	0	1	1	1	0
206	0,07	9,05	2,17	6	0,00	0	6	0	0	0	65,44	0	1	1	0	1	0	0	3	1	0,00	0,00	0	0	0	1	1	0
207	10,91	26,82	0,68	255	85,30	1	0	48	0	207	63,90	1	1	1	0	1	1	0	4	1	.	.	1	0	1	1	1	0
208	8,51	15,32	6,82	536	65,00	0	0	120	8	408	70,23	1	1	1	0	1	0	0	3	1	59,00	71,00	1	0	1	1	0	0
209	8,51	15,32	6,82	536	65,00	0	0	120	8	408	70,23	1	1	1	0	1	0	0	3	1	59,00	71,00	1	0	1	1	0	0
210	14,47	18,62	2,99	0	0,00	0	0	0	0	0	51,10	1	1	1	0	0	1	1	4	0	0,00	0,00	1	0	1	0	1	0
211	4,99	15,19	8,69	131	43,20	1	4	41	34	52	72,17	0	1	1	1	1	1	1	6	1	62,67	75,00	1	0	1	1	1	0
212	7,63	11,01	2,40	0	.	.	.	.	.	.	65,31	0	1	1	0	.	0	.	2	0	0,00	0,00	0	1	0	1	1	.
213	18,33	9,09	2,00	175	0,00	0	0	151	0	24	71,89	0	1	1	0	1	0	0	3	1	0,00	0,00	1	0	1	0	2	0
214	9,61	10,19	0,71	129	15,50	0	0	107	3	19	69,97	0	1	0	0	0	0	0	1	1	34,00	41,00	0	0	0	0	0	.
215	13,51	11,98	1,16	0	0,00	0	0	0	0	0	68,79	1	1	0	0	0	0	0	1	0	0,00	0,00	0	1	0	0	1	0
216	14,79	7,11	3,60	87	.	.	.	.	.	.	61,94	1	1	0	0	.	1	.	2	0	36,50	44,00	1	0	0	1	0	.
217	21,60	3,01	2,00	4	0,00	0	.	.	.	.	68,09	0	0	1	0	0	1	1	3	0	0,00	0,00	0	1	0	0	1	.
